# [^18^F]FDG PET-CT Imaging of the Low Back in Persistent Spinal Pain Syndrome Type 2: A Pilot Study Towards Improved Diagnosis

**DOI:** 10.3390/brainsci15070724

**Published:** 2025-07-07

**Authors:** Lara S. Burmeister, Richard L. Witkam, Kris C. P. Vissers, Martin Gotthardt, Dylan J. H. A. Henssen

**Affiliations:** 1 Department of Medical Imaging, Radboud University Medical Center, 6525GA Nijmegen, The Netherlands; lara.burmeister@radboudumc.nl (L.S.B.); martin.gotthardt@radboudumc.nl (M.G.); dylan.henssen@radboudumc.nl (D.J.H.A.H.); 2 Department of Anesthesiology, Pain and Palliative Medicine, Radboud University Medical Center, 6525GA Nijmegen, The Netherlands; jesper.witkam@radboudumc.nl; 3 Department of Nuclear Medicine, University Hospital Leipzig, 04103 Leipzig, Germany

**Keywords:** postoperative spinal pain, molecular imaging, chronic pain, biomarkers

## Abstract

**Background/Objectives**: Diagnosis of Persistent Spinal Pain Syndrome Type 2 (PSPS-T2) currently lacks objective biomarkers. Therefore, this retrospective study aimed to investigate differences in glucose metabolism in the axial musculoskeletal system in PSPS-T2 patients by means of [^18^F]FDG PET-CT imaging. **Methods**: Nine PSPS-T2 patients (five females, four males; mean age of 53 ± 4.82 years) and nine age- and gender-matched healthy controls (five females, four males; mean age of 53 ± 3.91 years) were included. For each participant, 24 regions of interest (ROIs) were manually drawn, including areas of the vertebral endplates, the intervertebral discs, and the psoas muscles. For each ROI, the mean standardized uptake values (SUVs) were assessed. Group differences were evaluated using repeated measures ANOVA with Bonferroni-adjusted post-hoc pairwise comparisons. Additionally, Pearson correlation analyses examined associations between SUV_mean_ values and the Numerical Rating Scale (NRS) pain scores. **Results**: Results demonstrated significantly higher SUV_mean_ values in healthy controls compared to PSPS-T2 patients, particularly at the superior endplates of L4 and S1, the intervertebral discs at L4-L5 and L5-S1, and the posterior endplates of L4 and L5. Although PSPS-T2 patients exhibited higher SUV_mean_ values than controls in the psoas muscle, these differences were not statistically significant. Additionally, no significant correlations were found between SUV_mean_ values and NRS pain scores, suggesting that metabolic activity alone does not directly reflect pain severity. **Conclusions**: Despite the limited sample size of this pilot study, the metabolic fingerprint of the axial musculoskeletal system was shown to be distinctly different in PSPS-T2 patients compared to healthy controls. This could lead to an improved understanding of PSPS-T2 pathophysiology and might open new doors for better diagnosis and treatment strategies.

## 1. Introduction

Persistent spinal pain syndrome type 2 (PSPS-T2) is characterized by persistent or emerging pain of the lower back and/or lower extremities despite anatomically successful surgery of the lower back or spine [1,2]. It is associated with significantly reduced health-related quality of life scores, often exceeding those seen in other chronic pain conditions [3,4]. Despite its high burden, PSPS-T2 remains challenging to diagnose [5].

Although radiological imaging remains essential to exclude serious underlying pathology, such as malignancy or fractions [6], the diagnostic value is limited in the majority of patients with chronic low back pain when no specific cause can be identified [7]. Instead, radiological imaging can even cause more harm than benefit by steering treatment algorithms in the wrong direction [8]. In many cases, symptom severity does not correlate well with structural abnormalities observed in imaging, which highlights the need for validated, phenotype-based approaches that better reflect the underlying pain mechanisms [9,10]. In this light, nuclear imaging techniques might be a promising example.

For instance, one study remarked that [^99m^Tc]HDP SPECT-CT imaging of the low back could help localize a potential pain generator [11]. In line with this, [^18^F]NaF PET-CT imaging was also found to be able to detect regions of increased osteoblastic activity among patients who had persistent or recurrent back pain following spinal fusion surgery, indicating possible pain-generating areas [12]. This was further investigated in PSPS-T2 patients by Peters et al. in 2015 [13] using [^18^F]NaF PET-CT imaging. In this study, significantly higher osteoblastic activity was observed in vertebral endplates in patients with the most severe clinical disability [13]. Sophisticated, osteoblastic activity imaging to identify possible bone-related pain-generating areas cannot be used alone to assess the multidimensionality of low back pain in PSPS-T2 patients. Previous studies demonstrated that changes in the (para)spinal muscles, such as atrophy or fatty infiltration, are associated with chronic low back pain [14]. Improved understanding of the biomechanics of low back pain has further highlighted the importance of muscular stabilization of the “neutral zone” range of motion in the low back by use of the (para)spinal muscles [15]. The neutral zone is thereby defined as the region where the spine can move with the greatest ease, with little influence from muscular control that usually comes into play when movement goes beyond this zone. When there is injury to the spine or weakness of the muscle, the zone may increase, which in turn can lead to spinal instability problems in the lower back [16]. In line with these insights, a recent study showed anatomic changes in the psoas muscles in PSPS-T2 patients on lumbar MRI scans, which correlated with various pain-related outcomes [17]. However, to our knowledge, no study has set out to investigate metabolic changes in the axial musculoskeletal structures, specifically in the lower back region, in PSPS-T2 patients.

Therefore, this study aimed to assess the difference in glucose metabolic activity in the vertebrae, disci, and psoas muscle between PSPS-T2 patients and healthy controls using [^18^F]FDG PET-CT scans.

## 2. Materials and Methods

### 2.1. Participants

Subjects were retrospectively included. To be included in this study, adult patients (≥18 years) diagnosed with PSPS-T2 must have undergone an [^18^F]FDG PET-CT with no signs of other pathologies that could have interfered with the measurements relevant to our outcome parameters (e.g., oncological diseases or infections). Another inclusion criterion was that patients must have received a spinal cord stimulation (SCS) device after the [^18^F]FDG PET-CT scan. This criterion ensured that all patients had a confirmed clinical indication for SCS implantation based on standard care, and that PET-CT was part of the diagnostic work-up without influencing the treatment decision. Including only patients who went on to receive SCS provided a homogeneous group with comparable disease severity and therapeutic trajectory. Additionally, nine age- and gender-matched controls were included. Controls were only included when [^18^F]FDG PET-CT showed no signs of possibly interfering with pathology. Indications for the controls to undergo [^18^F]FDG PET-CT imaging comprised the metabolic evaluation of incidentalomas in the lungs or adrenal glands previously observed on routinely performed anatomical imaging. Patients and controls who refused to share their data anonymously for scientific purposes were excluded from this study.

### 2.2. Pain Intensity Scores

Pain intensity was assessed using a numerical rating scale (NRS) ranging from 0 to 10 (i.e., 0 = no pain; 10 = worst imaginable pain). The NRS was obtained both prior to the SCS procedure and 12 months after the implantation of SCS.

### 2.3. [^18^F]FDG PET-CT Scan Acquisition

Scans were performed at the Department of Nuclear Medicine at the Radboudumc, Nijmegen, The Netherlands. A Siemens Biograph mCT PET UltraHD scanner equipped with a 40-slice adaptive 4D spiral CT was used. Prior to imaging, all participants were prepared with at least 6 h of fasting to ensure low blood glucose and low insulinemia, as this is directly responsible for glucose uptake by non-tumor cells. To ensure these conditions, blood glucose concentration was tested prior to scanning. To ensure optimal imaging conditions, the plasma glucose level needed to be lower than 10 mmol/L). The radionuclide fluorine-18 [^18^F], which was used in the form of F-18-fluorodeoxyglucose, was intravenously administered as a slow bolus injection. Administered activity was calculated following the recommendations of the European Associations of Nuclear Medicine when using a quadratic relationship between PET acquisition time per bed position and patient weight [18]. To ensure proper tracer distribution and to minimize residual activity, the intravenous line was flushed with 20 mL of 0.9% NaCl. Imaging was initiated 60 min post-injection.

### 2.4. [^18^F]FDG PET-CT Data Analysis

**Figure 1:**
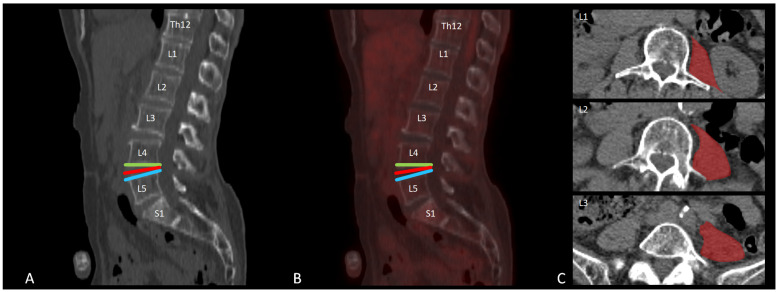
Example of a [^18^F]FDG PET-CT scan at the level of the lumbar spine of a non-painful individual to illustrate the regions of interest (ROIs) included in this study. (**A**) Sagittal low-dose CT reconstruction and (**B**) sagittal fused [^18^F]FDG-PET-CT image of the lumbar spine with ROIs at the level of L4–L5: superior endplate (green), intervertebral disc (red), and inferior endplate (blue). (**C**) Transversal low-dose CT images at three lumbar levels (L3–L5) with examples of ROI delineation (red) in the major psoas muscle.

Image annotation was carried out using a 3D slicer (https://www.slicer.org/). Regions of interest (ROIs) were drawn at the level of the superior and inferior endplates of the lumbar vertebrae (L1 to L5). Furthermore, ROIs were drawn at the level of the intervertebral discs from level T12/L1 to level L5/S1. Also, three ROIs were drawn at three levels (at level L3, L4, and L5) within the psoas major muscle on each side of the spine at transverse images; therefore, a total of 24 segments were examined for each participant (Figure 1).

In order to correct for the variability of glucose metabolism between subjects, two normalization methods of standardized uptake values (SUV_mean_ and SUV_max_) were employed. First, normalization was carried out by use of a volume of interest (VOI) located centrally in the normal-appearing liver parenchyma. Second, blood activity normalization was carried out by using a VOI in the lumen of the left cardiac ventricle.

### 2.5. Statistical Analysis

Statistical analyses were performed using SPSS (IBM Corporation, Version 29.0.2.0). Differences between groups across spinal levels were evaluated using repeated measures ANOVA to properly handle the repeated measurements within each subject. Following that, post-hoc pairwise comparisons were performed with Bonferroni correction to account for multiple comparisons. Statistical significance was set at *p* < 0.05, with all reported *p*-values adjusted using the Bonferroni method to control for the family-wise error rate. Pearson correlation analyses were performed to explore associations between NRS difference scores and SUV_mean_ values.

## 3. Results

Nine patients (five females, four males; mean age of 53 ± SD 4.82) and nine controls (five females, four males; mean age of 53 years; ±SD 3.91) were included. Patients underwent SCS implantation between 13 and 162 months after the [^18^F]FDG PET-CT imaging session.

The overall coefficients of variation were 49.5% and 63.1% when the SUV_mean_ values were normalized using the VOI placed in the liver and left ventricle, respectively. In contrast, when the SUV_max_ values were normalized in the same manner, the overall coefficients of variation were 55.0% and 73.4%, respectively. The SUV_mean_ values, which were normalized using the VOI placed in the liver, resulted in the lowest coefficient of variation. Therefore, these normalized SUV_mean_ values were used for further analyses.

Repeated measures analyses revealed statistically significant differences with higher scores in the control group regarding the SUV_mean_ for the superior endplates (F(1,16) = 6.76, *p* = 0.019), the discus (F(1,16) = 5.95, *p* = 0.027), and the posterior endplates (F(1,16) = 6.82, *p* = 0.019) between groups. Post-hoc Bonferroni-adjusted pairwise comparisons further specified these differences to the superior endplates of L4 (*p* = 0.048) and S1 (*p* = 0.037), the discs at L4-L5 (*p* = 0.043) and L5-S1 (*p* < 0.001), and the posterior endplates of L4 (*p* = 0.029) and L5 (*p* = 0.036), all showing higher SUV_mean_ values in the control group. These *p*-values, derived from Bonferroni-adjusted post-hoc pairwise comparisons, are detailed in Table 1. Higher SUV_mean_ values in the patient group were only found for the psoas muscle even though the repeated measures analysis for the psoas muscle revealed no statistically significant difference for neither the left psoas muscle (F(1,16) = 0.59, *p* = 0.453) nor the right psoas muscle (F(1,16) = 0.17, *p* = 0.686). Pearson correlation analyses revealed no statistically significant correlations between the NRS difference scores and the SUV_mean_ values.

## 4. Discussion

The present study revealed higher metabolic activity in the region of the lower back in non-painful controls as compared to PSPS-T2 patients. More specifically, SUV_mean_ values were significantly higher in the control group at the superior endplates of L4 and S1, the intervertebral discs at L4-L5 and L5-S1, and the posterior endplates of L4 and L5. We found no significant correlation between the mean SUV values and pain intensity scores. These preliminary results could help provide insights into the biological mechanisms that might lead to PSPS-T2 at the level of the lower back.

Generally, a rise in glucose metabolism in the vertebral structures suggests enhanced metabolic activity, which could be related to bone remodeling processes such as inflammation or degeneration [19,20,21]. However, the lower SUV_mean_ values in the PSPS-T2 patients may suggest an alternative pathological mechanism accompanying this condition. One possible explanation for the observed metabolic differences could be the presence of fibrotic changes. Fibrosis is driven by activated fibroblasts, which play a role in various pathological processes such as scarring, degeneration, and inflammation [22]. Epidural fibrosis, characterized by epidural fat being replaced with scar tissue, is a well-known complication following spinal surgery [23]. Previous research has found a positive association between the number and extent of spinal surgeries and the incidence of fibrosis, as well as a significant relationship between the severity of patients’ symptoms and the extent of fibrosis [24]. Furthermore, intervertebral disc fibrosis is known to play an important role in intervertebral disc degeneration [25,26]. As fibrosis has been described to elicit a homogeneous, mild FDG-uptake [27], the lower SUV_mean_ values in the patient group may be explained by the presence of fibrotic tissue that is less metabolically active compared to the normal physiological uptake that is seen as a consequence of the axial load on bones, ligaments, and muscles. A possible explanation for our findings could thus be that PSPS-T2 patients have a higher degree of spinal and intervertebral disc fibrosis, which may not be adequately captured by [^18^F]FDG PET-CT imaging.

Fibroblast-activation protein (FAP) is expressed by activated fibroblasts in epidural fibrosis and can be utilized for nuclear imaging [22]. It has been shown that FAP-specific imaging, such as [^68^GA]FAPI PET-CT, provides a better reflection of fibrosis progression compared to [^18^F]FDG PET-CT [28]. FAP-specific imaging could hence be a promising alternative to [^18^F]FDG PET-CT in exploring the role of fibrosis in PSPS-T2.

The iliopsoas muscle is hypothesized to play a crucial role in stabilizing the lumbar spine, and previous studies demonstrated a correlation between its cross-sectional area and decreased pain intensity in individuals experiencing lower back pain [29]. In contrast to previous findings that indicated metabolic changes in the psoas muscle in PSPS-T2 patients [17], we were unable to identify statistically significant differences in glucose metabolism in this muscle group when comparing PSPS-T2 patients to healthy controls. As demonstrated by several studies, low back pain is frequently accompanied by structural changes of the lumbar muscles [30,31]. These alterations consist of muscular atrophy and the infiltration of fat, which are also known to be correlated with inactivity [32,33] Interestingly, there seems to be a reversed relationship in changes between the posterior paraspinal muscles and the psoas muscle, where a higher fat infiltration in the posterior paraspinal muscles, such as the multifidus, is associated with a lower infiltration of fat in the psoas muscle. It is proposed that this could represent a compensating mechanism in which the psoas compensates for the loss in strength in order to stabilize the spine [34,35]. Our findings suggest that even though structural alterations such as atrophy and fatty infiltration may occur, they do not necessarily correspond with increased metabolic activity as shown by [^18^F]FDG PET-CT. Nonetheless, interpretation of these findings necessitates careful consideration of the movement and activity profiles of both PSPS-T2 patients and healthy controls, as disparities in physical activity may confound the observed metabolic patterns. To enhance the validity of metabolic comparison across cohorts, future studies should include objective assessments of physical activity and further investigate these pathways by integrating additional muscle groups and alternative imaging modalities such as MRI.

As the absence of a significant correlation between SUV_mean_ values and pain intensity scores suggests, metabolic activity alone may not be a direct reflection of the severity of pain in PSPS-T2 patients. This aligns with the broader understanding of chronic pain, which assumes that chronic pain can be seen as a multifactorial condition that is influenced by a variety of mechanisms such as neuroinflammatory processes, central sensitization, and altered processing in the central nervous system [36,37,38]. A possible explanation for the lack of correlation could be the relatively small sample size and the long period over which patients were included in the study. Given that metabolic processes such as inflammation and remodeling evolve over time, it could be possible that some of these changes may have been stabilized by the time of scanning, making them less detectable on [^18^F]FDG PET-CT imaging. Furthermore, several cognitive behavioral and psychosocial factors, such as anxiety, pain catastrophizing, depression or a person’s coping mechanisms, play an important role in the perception of pain [39,40,41]. In addition to the localized metabolic changes in the spine, all of these mechanisms could have the potential to modulate the experience of pain.

### Limitations

When interpreting the current study’s findings, there are several limitations that should be considered. Firstly, the study sample consists of a limited number of participants, which may constrain the extent to which the results can be generalized. Secondly, given the retrospective nature of the study design, it is not possible to assess metabolic changes over time, such as assessing changes before and after SCS implantation or following other major events such as additional surgeries of significant life changes that could influence pain perception or behavior and thereby affect PET imaging outcomes. The conduction of prospective studies that assess the impact of SCS on glucose metabolism may offer further insights into the mechanisms underlying the treatment response. Additionally, even though [^18^F]FDG PET-CT is indicative of metabolic activity, it is not possible to differentiate between inflammatory, reconstructive, or degenerative processes. Integrating findings from additional biomarkers such as [^68^GA]FAPI PET-CT or the MRI-based assessment of muscle integrity could further enhance our understanding of the underlying pathophysiology in PSPS-T2.

## 5. Conclusions

This pilot study explores a potential role for metabolic imaging in identifying pain-related changes with differences in glucose metabolic activity observed in vertebral structures, particularly the endplates and discs, between patients with PSPS-T2 and controls. The absence of significant findings in psoas metabolism and correlations with pain scores may reflect the multifactorial nature of chronic pain in PSPS-T2. Future research should therefore explore multi-modal imaging approaches, combined with psychological assessments, to investigate the interactions between structural, metabolic, and neuropsychological changes in this patient population with dynamic biopsychosocial interactions.

## Figures and Tables

**Table 1:** Overview of the SUV_mean_ values and standard deviations per group and region.

	Level	Patients	Controls	*p*-Value *
Superior endplates	L1	0.55 (0.15)	0.71 (0.38)	0.266
	L2	0.56 (0.17)	0.75 (0.31)	0.131
	L3	0.56 (0.20)	0.78 (0.31)	0.097
	L4	0.55 (0.17)	0.80 (0.29)	0.048
	L5	0.60 (0.19)	0.87 (0.37)	0.074
	S1	0.57 (0.21)	0.78 (0.20)	0.037
Discus	L1–L2	0.40 (0.10)	0.52 (0.21)	0.152
	L2–L3	0.39 (0.20)	0.47 (0.10)	0.293
	L3–L4	0.40 (0.24)	0.55 (0.20)	0.182
	L4–L5	0.49 (0.26)	0.71 (0.15)	0.043
	L5–S1	0.40 (0.14)	0.75 (0.21)	<0.001
	T12–L1	0.44 (0.11)	0.61 (0.31)	0.134
Psoas left	L3	0.58 (0.51)	0.43 (0.14)	0.414
	L4	0.55 (0.48)	0.41 (0.15)	0.414
	L5	0.49 (0.39)	0.41 (0.12)	0.573
Psoas right	L3	0.60 (0.57)	0.39 (0.11)	0.301
	L4	0.55 (0.55)	0.44 (0.13)	0.547
	L5	0.46 (0.34)	0.57 (0.29)	0.446
Posterior endplates	L1	0.59 (0.13)	0.67 (0.29)	0.451
	L2	0.57 (0.15)	0.66 (0.23)	0.331
	L3	0.56 (0.12)	0.78 (0.37)	0.104
	L4	0.58 (0.16)	0.85 (0.30)	0.029
	L5	0.53 (0.14)	0.79 (0.31)	0.036
	S1	0.59 (0.17)	0.77 (0.36)	0.195

* Adjustment for multiple comparisons: Bonferroni.

## Data Availability

The datasets generated and analyzed during the current study are available from the corresponding author on reasonable request. The data are not publicly available due to their containing information that could compromise the privacy of the participants.

## Abbreviations

The following abbreviations are used in this manuscript:

PSPS-T2	Persistent Spinal Pain Syndrome Type 2
[^18^F]FDG PET-CT	Fluorine-18 Fluorodeoxyglucose Positron Emission Tomography-Computed Tomography
ROI	Region of interest
SUV	Standardized uptake value
NRS	Numerical Rating Scale
[^99^mTC]HDP SPECT-CT	Technetium-99m Hydroxymethylene Disphosphonate Single Photon Emission Computed Tomography-Computed Tomography
[^18^F]NaF PET-CT	Fluorine-18 Sodium Fluoride Positron Emission Tomography-Computed Tomography
SCS	Spinal cord stimulation
NaCl	Sodium Chloride
VOI	Volume of interest
FAP	Fibroblast-activation protein
[^68^Ga]FAPI PET-CT	Gallium-68 Fibroblast Activation Inhibitor Positron Emission Tomography-Computed Tomography
